# Linear response theory in stock markets

**DOI:** 10.1038/s41598-021-02263-6

**Published:** 2021-11-29

**Authors:** Antonio M. Puertas, Juan E. Trinidad-Segovia, Miguel A. Sánchez-Granero, Joaquim Clara-Rahora, F. Javier de las Nieves

**Affiliations:** 1 Departamento de Química y Física, Universidad de Almería, 04.120 Almería, Spain; 2 Departamento de Economía y Empresa, Universidad de Almería, 04.120 Almería, Spain; 3 Departamento de Matemáticas, Universidad de Almería, 04.120 Almería, Spain; 4 KHN Capital Consulting, Avda. Diagonal 640 6th floor, 08017 Barcelona, Spain

**Keywords:** Statistical physics, Nonlinear phenomena

## Abstract

Linear response theory relates the response of a system to a weak external force with its dynamics in equilibrium, subjected to fluctuations. Here, this framework is applied to financial markets; in particular we study the dynamics of a set of stocks from the NASDAQ during the last 20 years. Because unambiguous identification of external forces is not possible, critical events are identified in the series of stock prices as sudden changes, and the stock dynamics following an event is taken as the response to the external force. Linear response theory is applied with the log-return as the conjugate variable of the force, providing predictions for the average response of the price and return, which agree with observations, but fails to describe the volatility because this is expected to be beyond linear response. The identification of the conjugate variable allows us to define the perturbation energy for a system of stocks, and observe its relaxation after an event.

## Introduction

Linear response theory (LRT)^1^, allows resolving the response of a system to a weak external perturbation considering the dynamics of the system at equilibrium subjected to fluctuations. This is a practical extension of Onsager’s regression hypothesis, namely, a system relaxes to equilibrium after an external perturbation in a similar manner as from fluctuations^2^. This powerful tool and the formalism of time correlation functions have been applied to study several physical systems, such as soft matter^3–6^, spin glasses^7^, or magnetism^8^, but also it has been used to derive a conceptual basis for equilibrium and non-equilibrium thermodynamics^9,10^. The drawback is that only the first order in the perturbation is retained, which might not be sufficient in some cases^11^.

In LRT, a linear perturbation to the equilibrium Hamiltonian of the system is assumed, $H = H_0 + AF$, where $H_0$ denotes the Hamiltonian in the equilibrium (non-perturbed) state, and $H'=AF$ the perturbation, with *F* the external force, which is conjugate to the variable *A*. The theory restricts to small forces, and states that the change in a variable *B*(*t*) due to the application of the force is given by^1,12^:

1 $$\begin{aligned} \langle \Delta B (t) \rangle = \int _{-\infty }^t \Phi _{BA}(t-t') F(t') dt' \end{aligned}$$

where the after-effect function $\Phi _{BA}(s)$ is set by the correlation function:

2 $$\begin{aligned} \Phi _{BA}(s)\,=\, \beta \langle B(s) \dot{A} \rangle = - \beta \langle \dot{B}(s) A \rangle \end{aligned}$$

which is calculated in the unperturbed (equilibrium) state, with $\beta$ the inverse thermal energy, and $\dot{A}$ denotes the time derivative of *A*. LRT, both in the classical and quantum forms, have been applied mainly to the calculation of transport coefficients in several systems, such as colloids, charge transport, ferromagnetization or liquid crystals^13^, but also in other more exotic fields, such as neurophysiology^14^ or climate science^15,16^. In this paper, we aim to apply LRT to a very different field, namely, stock markets.

The application of physical theories and models to financial markets has attracted interest since the work of Bachelier in 1900^17^, and in particular in the last three decades^18–21^. Most models or applications describe financial market dynamics as equilibrium systems subjected to fluctuations, fulfilling the fluctuation–dissipation theorem^22^, such as a Brownian particle or system^23–26^, while other works try to analyze its entropy or complexity^27,28^. The non-Gaussianity of fluctuations causing anomalous diffusion has also received attention from the early works of Mandelbrot^29,30^, where elaborate models to describe such fluctuation distributions have been resolved, e.g., by considering truncated Levy flights^31,32^, the Tsallis entropy model^33,34^, hopping in the free energy landscape in glasses^35–37^, or extending the continuous-time random walk model^38,39^.

Within a Physics scope, regime switching models have been applied to market dynamics^40^. Such transition is straightforward for instance in changes in economic policy, such as the Quantitative Easing policies from the European Central Bank (ECB) and the Federal Reserve Board (FED)^41,42^, or abrupt when unexpected events occur, such is the case of the Great Recession from 2008, or the still ongoing crisis due to the spread of the COVID-19 pandemics^43–45^. Such regimes are characterized according to economic and business cycles of expansion and recession^46^. There, different classes of random walks have been identified, where the nature of price changes are resolved not due to the unpredictable nature of incoming news but a direct consequence of competition between market forces led by liquidity and market takers and makers^47^. Long-range correlated market orders and activity lead to diffusive and super-diffusive dynamics, while mean reverting limit orders determine sub-diffusive market conditions^36,47^. In such framework, the linear response formalism has been considered when studying casual relations in markets, where characteristic volatility and stock dynamic regimes are identified as influencing the overall market dynamics prior to financial crashes, while individual volatility of securities follow collective market behavior after the crash event^48^. Moreover, the breakdown of linear response has been found in periods of low market liquidity and transaction, where fluctuations become large enough so that market dynamics is strongly displaced from equilibrium, and second or larger order energy terms must be accounted for in the Hamiltonian of the system^49^.

Our aim in this work is to apply LRT to a system of stocks, thus enlarging the applicability of LRT and also advancing in the knowledge of the mechanisms governing the stock dynamics. For this purpose, a given financial market is assumed to be an equilibrium system subjected to fluctuations due to its internal dynamics, and perturbed by external forces. Within LRT we attempt to study weak forces, where the effects are linear with force strength. LRT can provide then the evolution of the system after the application of the external force. Thus, for this analysis the following steps have been followed: (1) measurement of the response of the system after the application of an external perturbation, (2) identification of the variable *A*(*t*), conjugate to the force, and (3) calculation of the response function according to LRT, to finally compare it with the “empirical” function obtained in (1). As a final result, in addition to the extension of LRT, the perturbation energy in a stock market can be defined. Note that since we do not base the identification of the variable conjugate on a physical model, we only rely only on the validity of LRT for stock markets.

A database of 862 stocks has been used, corresponding to the companies in the NASDAQ index from 03/01/2000 to 30/10/2020. The “Supplementary Information” to this article provides a similar analysis for a set of European stocks and the NYSE, yielding similar results.

## Results

Consider a charged colloidal particle in water: internal forces are caused by thermal and density fluctuations in the solvent and provoke the particle Brownian motion, whereas external forces can be caused by electric or gravitational fields^50^. Stock prices, on the other hand, are set by brokers or other practitioners, according to supply and demand, as well as to their investment strategies and expectations; these can be considered as internal forces. However, there are factors that strongly influence market prices, such as political decisions, announcements of results, companies acquisition or merger, bankrupts, ... These can be considered as external forces, which, different from the physical counterpart, act onto the stock prices through the same practitioners as the internal forces. This ambiguous recognition of external forces poses a major problem on their identification, as well as its strength scale, and the conjugate variable *A*(*t*), needed for the application of the LRT formalism. In fact, it is generally accepted that only a fraction of the motion of stocks can be attributed to fundamental economic information that could have had a pronounced impact on cash flow forecasts or discount rates^51,52^.

**Figure 1:**
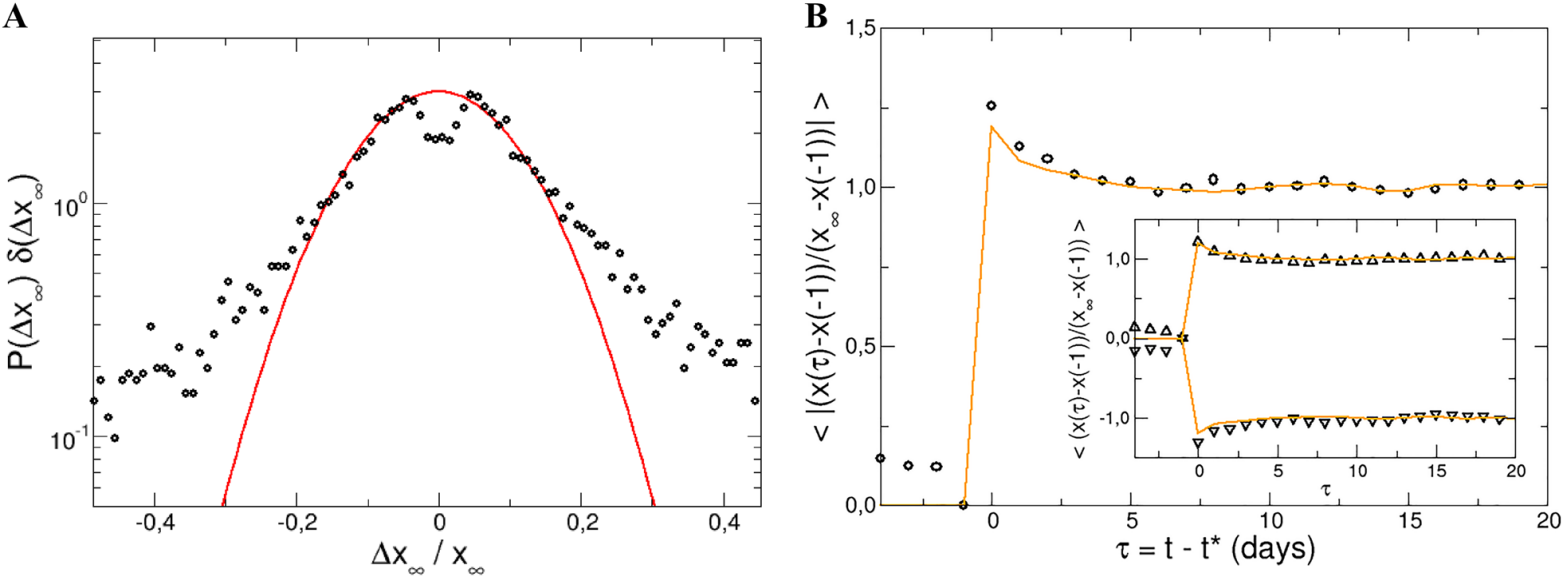
Analysis of events. Left panel (**A**) distribution of the relative total log-price variation (circles), with a Gaussian fitting to the maximum (red line). Right panel (**B**) evolution of the log-price before and after small events in absolute value (circles). The inset shows positive and negative events separatedly. The lines are the prediction from LRT (see text below).

Therefore, we do not make any assumptions concerning external forces, and adopt a phenomenological point of view following previous works on events^53^: a dramatic event, assumed to be provoked by an external force, takes place whenever the absolute value of the one day log-return of a stock surpasses four times the root mean square deviation of log-returns of this stock. This threshold for the definition of an event is arbitrary but in line with previous studies^54^, as it allows the segmentation of events in equilibrium fluctuations or dramatic perturbations. In any case, its specific value has little effect on the results presented below, as far as it is well above 1. In the following, we assume that the external force starts to act at the event time $t^*$, and keeps acting indefinitely, or until a new event takes place. Furthermore, we assume that the impact of different forces are well separated, i.e. the evolution of a stock price after a force is applied relaxes to equilibrium before a new force acts; thus events separated less than 10 days are discarded. With such criteria, ca. 5000 events are identified in the whole set ($\sim 2300$ positive events, with positive log-return $v(t^*)>0$, and $\sim 2700$ negative ones, with $v(t^*)<0$). Note that very dramatic events, such as the financial crisis in 2008 or the COVID19 pandemic in 2020, provoke drastic changes in the price extending over several days^54,55^, and are excluded from our analysis according to this selection, because LRT is expected to fail for large external forces. The resulting distribution of events per day (affecting different stocks) is a decreasing function, with 75% of the events in days with less than five events, which guarantees that the events are indeed independent. Fig. 1 analyzes the stock log-price after an event, namely the response function of the log-price after an event”.

Figure 1A presents the distribution of the total log-price variations provoked by the event, namely $\Delta x_{\infty } = x_{\infty }-x(t^*-1)$, where $x(t^*-1)$ is the log-price just before the event, and $x_{\infty }$ is the log-price well after the event. The local minimum at $\Delta x_{\infty }=0$ is caused by our definition of events, and disappears if a smaller threshold is selected. On the other hand, the distribution displays positive deviations from Gaussian behaviour for price differences above $0.15 x_{\infty }$ in absolute value. These deviations are typical in finance, and have been the topic of intense research and debate^21^. To our purpose, the deviation from the Gaussian profile serves to identify “small” and “large” events, and therefore determine the expected validity range of LRT. All subsequent analysis is restricted to small events. In Fig. 1B the mean normalized log-price evolution around the event in absolute value is presented. This shows an overshoot at $t^*$, namely when the event takes place, followed by a decay within a few days to reach a steady value ($x_{\infty }$ is the average log-price between 10 and 20 days after the event). The inset shows positive and negative events separatedly.

**Figure 2:**
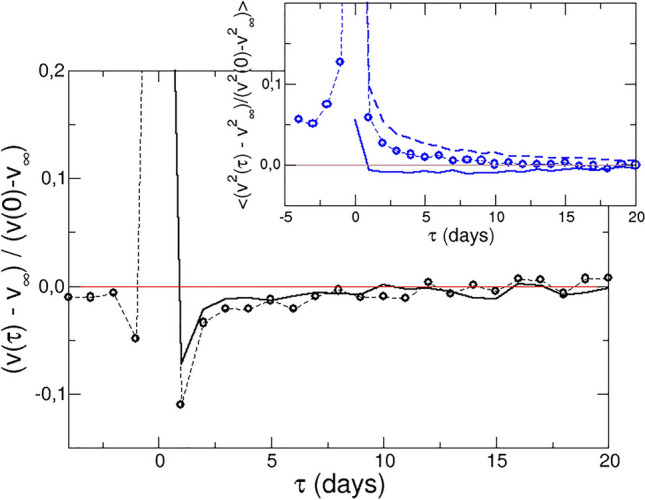
Evolution of the log-return and the volatility (inset) before and after small positive and negative events. The lines show the prediction of linear response theory (see text below).

In addition to the log-price, the dynamics of stocks is also monitored considering the corresponding log-return and volatility. (The latter represents how fast the price changes, irrespective of the sign of the change, and is calculated as $v^2_i(t)$). The average normalized evolution of the one day return and volatility after an event are shown in Fig. 2. The average log-return for a positive (negative) event increases (decreases) at the event, and decreases (increases) abruptly immediately after it, followed by a slow relaxation to the equilibrium value. The figure represents the normalized evolution averaged for positive and negative events. The volatility, on the other hand, increases at $t^*$ and then decreases to the “equilibrium” magnitude at both positive and negative events.

Figures 1 and 2 show that the evolution of the log-price, log-return and volatility is abrupt at the event, and then relaxes to a steady value for a few days. From a physical point of view, this indicates that these variables display memory, and according to LRT, the correlation functions with variable *A*(*t*), conjugate of the force, should decay with a time scale of a few days. In order to identify this variable, the log-price, log-return and volatility autocorrelation functions (ACF) have been studied (see “Methods”).

**Figure 3:**
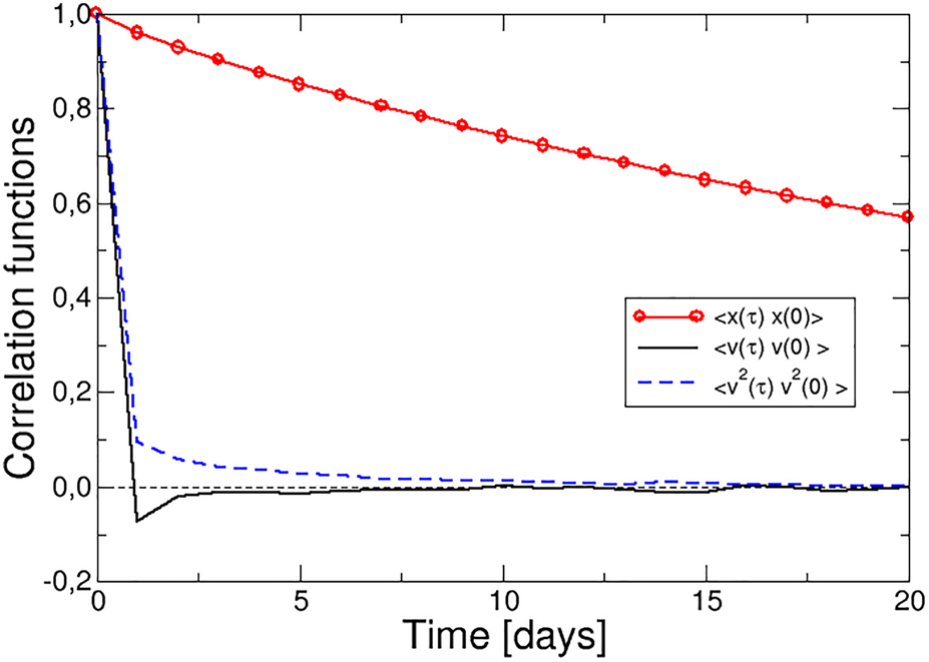
Autocorrelation functions of the log-price (red line and circles), log-return (continuous black line) and volatility (broken blue line).

They are presented in Fig. 3 and show very different behaviour: whereas the log-return reaches negative values within the first day and then relaxes to zero (resembling the velocity ACF in hard spheres), and the volatility ACF presents a similar time scale, but a monotonous decay, the time scale of the log-price ACF is $\sim 50$ days. For the purpose of applying LRT, the log-return is more appropriate due to the similarity with the time scale and behaviour of its response function. Therefore, we tentatively identify the log-return, *v*(*t*), as the conjugate variable, *A*(*t*), to the external force. The response of the log-return is therefore provided directly by its ACF, assuming that the force follows a Heaviside functional form, $F(t)=F_0 \theta (t )$ as discussed above:

3 $$\begin{aligned} \langle \Delta v(\tau ) \rangle = - \beta \int _0^{\tau } F_0 \langle \dot{v}(\tau ') v(0) \rangle d\tau '= - \beta F_0 \langle v(\tau ) v(0) \rangle \end{aligned}$$

Since the strength of the force is unknown, we plot $C\left( v,v\right)$ to compare with the normalized response of the log-return in Fig. 2 (black line). The prediction from LRT agrees with the empirical response function.

Once the conjugate variable, *A*(*t*) in Eq. (1), has been identified as the log-return, the average evolution of other variables can be readily obtained using LRT. In particular, for the log-price, the integral of the log-return ACF above provides the predicted response, according to LRT:

4 $$\begin{aligned} \langle \Delta x(\tau ) \rangle = - \beta F_0 \int _0^{\tau } \langle v(\tau ') v(0) \rangle d\tau ' \end{aligned}$$

since $\dot{x}=v$. This is included also in the right panel of Fig. 1. Again, good agreement between this prediction and the observations is found. Similar comparisons between the predictions from LRT and the evolution of the log-price and log-return for a set of European stocks and for the NYSE are presented in Figs. 1 and 3 of the “Supplementary Information”. Note that in any case, LRT predicts the average response of the variable, and cannot be used to calculate the evolution of a single stock (or in physical terms, of a single trajectory in phase space).

The volatility, on the other hand, is a second-order variable and it is not expected that it can be described within LRT. This is tested in the inset to Fig. 2, where the cross correlation function $\langle v^2(\tau ) v(0) \rangle$ is included (continuous line), as well as the volatility ACF (broken line). None of them correctly describes the observed evolution of the volatility, although the predction from LRT (continuous line), captures qualitatively the slow decay of the volatility after the event.

For constant external forces, LRT also provides the coupling constant of the system in the stationary regime as the integral of $\Phi _{BA}(t)$ extended to $+\infty$, i.e., $\langle \Delta B_{\infty } \rangle = k F_0$. For the case of autocorrelation functions, these constants are the transport coefficients associated with the flux induced by the external force, and depict the Green–Kubo relations^56^. In our case, two coefficients can be calculated:

5 $$\begin{aligned}&k_x = - \int _0^{\infty } \langle v(\tau ) v(0) \rangle d\tau = - 1.25 \times 10^{-3} \nonumber \\&k_v = \langle v(0) v(0) \rangle - \lim _{\tau \rightarrow \infty } \langle v(\tau ) v(0) \rangle = 1.55\times 10^{-3} \end{aligned}$$

Note that the correlation function $\langle v(\tau ) v(0) \rangle$ is used here, instead of the normalized *C*(*v*, *v*) used above.

**Figure 4:**
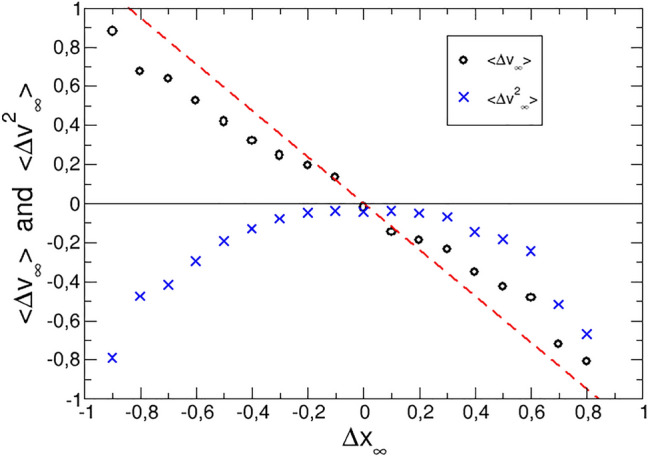
Mean log-return and volatility variations as a function of the total log-price variation for small events, as labelled. The solid red line indicates the prediction for the log-return.

To test these results, we display in Fig. 4 the average total variation of the log-return, $\langle \Delta v_{\infty } \rangle = \langle v_{\infty }-v(0) \rangle$ as a function of the log-price total variation, $\langle \Delta x_{\infty } \rangle$ for small events. The expected linear relationships with the force, yield $\langle \Delta v_{\infty } \rangle = k_v/k_x \,\, \langle \Delta x_{\infty } \rangle$, which is also included in Fig. 4. The data show good agreement with the predictions, particularly for small price variations, where the theory is expected to perform better. For the volatility, the total variation has been also included in the figure as a function of the log-price variation, but the dependence is clearly not linear.

**Table 1:** Values of the coefficients $k_x$ and $k_v$ for the three sets of stocks considered.

Stock market	$k_x$	$k_v$
NASDAQ	$-12.5\times 10^{-4}$	$15.5\times 10^{-4}$
NYSE	$-15.3\times 10^{-4}$	$25.7\times 10^{-4}$
European	$-3.1\times 10^{-4}$	$3.6\times 10^{-4}$

Table 1 presents the results of the coefficients $k_X$ and $k_v$ for the NYSE and European sets of stocks, studied in more detail in the “Supplementary Information”. The concomitant tests of the linearity of $\langle \Delta v_{\infty } \rangle$ vs. $\langle \Delta x_{\infty } \rangle$ are also presented there. Note that $k_x$ and $k_v$ are much larger (in absolute value) for the NASDAQ and NYSE than for the European stocks, implying that the European set is less affected by external forces, probably due to its heterogeneity.

Once variable *A* has been identified as the log-return, the perturbation energy can be calculated if the force is known. Nevertheless, within the linear regime, the total log-price variation is proportional to the force, $\Delta x_{\infty } = k_x F$, and the energy can be calculated as:

6 $$\begin{aligned} H'(\tau ) = \frac{1}{k_x} \Delta x_{\infty } v(\tau ) \end{aligned}$$

The distribution of perturbation energies shows a symmetric bell shape with the expected wings or tails for large (positive and negative) values. Since for both positive and negative events the product $\Delta x_{\infty } \Delta v(\tau )$ is positive at the event, the sign of $k_x$ determines if the energy is positive or negative for both types of events. Our calculations yield a negative $k_x$, which corresponds to a negative perturbation energy, with respect to the value just before the event.

**Figure 5:**
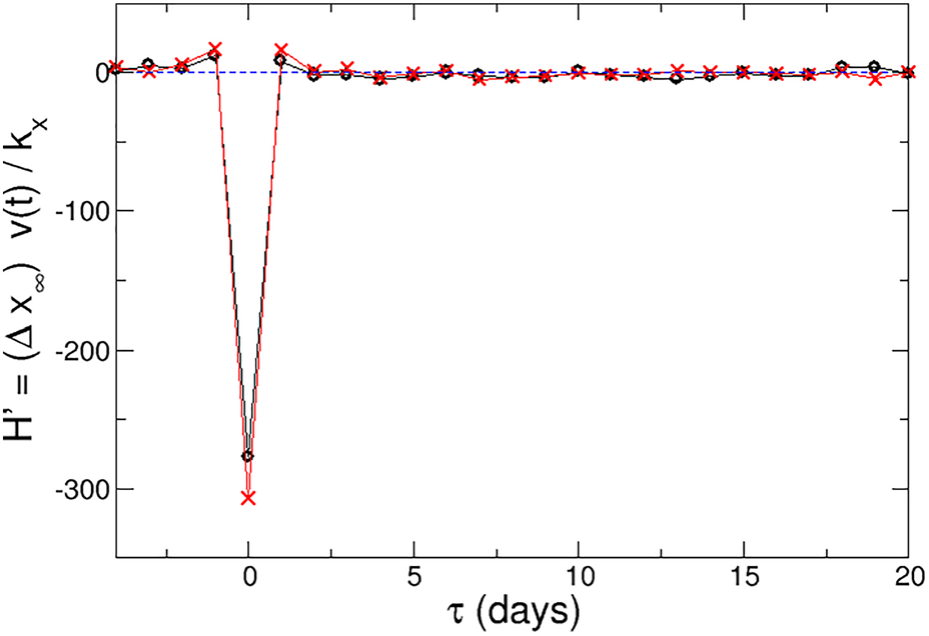
Perturbation energy for positive (black circles) and negative events (red crosses).

Figure 5 presents this energy for both kinds of events. The energy is near zero before and well after the event, when its effect has dissipated, but grows (in absolute value) notably for all events. This effect dissipates as equilibrium is recovered. From a physical perspective, this is equivalent to a system where the energy input dissipates and the system returns to equilibrium. The time scale for the dissipation is the same as for the decay of the log-return ACF, as the force is continuously active for $t>t^*$. Interestingly, the figure indicates that the perturbation energy is also non-zero for $t<t^*$, i.e., before the event. This is beyond our current interpretation of the results, where the force can only affect the system for positive times, but could be tackled with a time dependent force. Also, such feature could serve as an indicator of an event in the next few days. Nevertheless, it must be recalled that our modeling considers averages over many different events in 20 years and a set of ca. 850 stocks. Thus, predicting events to a single stock is far beyond the purpose of this work.

## Discussion

We have resolved Linear Response Theory as an efficient framework to determine the response of a system such as the stock market, which is indeed hallmarked by fluctuations. The autocorrelation functions of the log-price, log-return, and volatility indicate that the most appropriate variable to be considered conjugate of the external force is the log-return, due to its relaxation kinetics. Thus, the predicted response functions for the log-price and log-return have been calculated and agree with the results obtained from the empirical analysis of stock prices. Both of them show an overshoot at the event, and a slower recovery towards equilibrium within 2–3 days, in resemblance with the behaviour of a dissipative system. The identification of the energy in a stock market represents a major goal, strikingly supported on a well-established physical ground.

The results presented here have been obtained for the NASDAQ, extending over the last 20 years considering the stocks that have belonged continuously to the index. Similar results have been also obtained for a set of European national floors, although the statistics is much better in the case of the NASDAQ, and New York Stock Exchange. These results provide further support of the results and conclusions presented here.

In any case, we stress that there is no physical model supporting this identification of the log-return as conjugate to the external force. The results presented here are based on a phenomenological approach, but show the compatibility of financial markets with well-established physical theories, as far as an appropriate analogy of variables is performed.

## Methods

All stocks used for this study have been taken from Yahoo! Finance, with a time resolution of 1 day. The databases have been comprised by all stocks that have belonged continuously to the given market. For the NASDAQ (main text) and NYSE (“Supplementary Information”), stocks that have been active from 03/01/2000 to 30/10/2020 were selected, amounting to 862 stocks and 1084, respectively. For the european stocks, the set of stocks is constructed with companies that have belonged continuously to the national indices of the UK (FTSE100), Germany (DAX30), France (CAC40), Spain (IBEX35), Switzerland (SMI), Italy (FTSE MIB), Portugal (PSI20), and Holland (AEX). This set comprises 240 stocks, corresponding to big and stable European companies, sampled every day since 2010–2019.

As usual in financial studies, we consider the logarithm of the price, termed log-price, $x_i(t)$ and only working days in the analysis, i.e. weekends are not taken into account. The one-day log-return is defined as $v_i(t)=x_i(t)-x_i(t-1)$ and the volatility is calculated as $v_i^2$.

A dramatic event, assumed to be provoked by an external force, takes place whenever the absolute value of the one day log-return of a stock surpasses four times the root mean square deviation of log-returns of this stock, i.e., if:

7 $$\begin{aligned} |v_i(t^*)| = |x_i(t^*)-x_i(t^*-1)| \ge 4 \sqrt{ \frac{1}{n_t-1} \sum _{j=2}^{n_t} v_i^2\left( t_j\right) } \end{aligned}$$

where $n_t$ is the total number of days in our sample.

The time auto-correlation function between the discrete variables *X* and *Y*, $\left\{ X_i\right\} = \left\{ X(t_i)\right\}$ and $\left\{ Y_i\right\} =\left\{ Y(t_i)\right\}$, with $i=1,\ldots N$, is calculated as:

$$\begin{aligned} C(X,Y) = \frac{\sum X_i Y_i - N \bar{X} \bar{Y}}{(N-1) S_x S_y} \end{aligned}$$

where $\bar{X}$ and $S_x$ stand for the sample mean and standard deviation, respectively.

The non-normalized correlation function, $\langle X Y \rangle$, has also been used to calculate the coefficients $k_x$ and $k_v$. This is defined as:

$$\begin{aligned} \langle X Y \rangle = \frac{\sum X_i Y_i - N \bar{X} \bar{Y}}{N^2} \end{aligned}$$

## Supplementary Information

Supplementary Information.

## References

[1] Kubo R (1957). Statistical-mechanical theory of irreversible processes. I. General theory and simple applications to magnetic and conduction problems. J. Phys. Soc. Jpn..

[2] Onsager L (1931). Reciprocal relations in irreversible processes. I. Phys. Rev..

[3] Benetatos P, Frey E (2004). Linear response of a grafted semiflexible polymer to a uniform force field. Phys. Rev. E.

[4] Boschan J, Vasudevan SA, Boukany PE, Somfai E, Tighe BP (2017). Stress relaxation in viscous soft spheres. Soft Matter.

[5] Vogel F, Zippelius A, Fuchs M (2019). Emergence of Goldstone excitations in stress correlations of glass-forming colloidal dispersions. Europhys. Lett..

[6] Dal Cengio S, Levis D, Pagonabarraga I (2019). Linear response theory and Green–Kubo relations for active matter. Phys. Rev. Lett..

[7] Djurberg C, Mattsson J, Nordblad P (1995). Linear response in spin glasses. Europhys. Lett..

[8] Swiecicki SD, Sipe JE (2014). Linear response of crystals to electromagnetic fields: Microscopic charge-current density, polarization, and magnetization. Phys. Rev. B.

[9] Bianucci M, Mannella R, West BJ, Grigolini P (1995). From dynamics to thermodynamics: Linear response and statistical mechanics. Phys. Rev. E..

[10] Nazé P, Bonança MVS (2020). Compatibility of linear-response theory with the second law of thermodynamics and the emergence of negative entropy production rates. J. Stat. Mech..

[11] Van Vliet CM (1988). On van Kampen’s objections against linear response theory. J. Stat. Phys..

[12] Hansen J-P (2006). Theory of Simple Liquids.

[13] Forster D (1975). Hydrodynamic Fluctuations, Broken Symmetry, and Correlation Functions (Frontiers in physics.

[14] Cessac B (2019). Linear response in neuronal networks: From neurons dynamics to collective response. Chaos.

[15] Leith CE (1975). Climate response and fluctuation dissipaction. J. Atmos. Sci..

[16] Bódai T, Lucarini V, Lunkeit F (2020). Can we use linear response theory to assess geoengineering strategies?. Chaos.

[17] Bachelier L (2006). Theory of Speculation.

[18] Stanley HE (1996). Anomalous fluctuations in the dynamics of complex systems: From DNA and physiology to econophysics. Proceedings of 1995 Calcuta conference on dynamics of complex systems. Physica A..

[19] Stanley HE, Amaral LAN, Canning D, Gopikrishanan P, Lee Y, Liu Y (1999). Econophysics: Can physicists contribute to the science of economics?. Physica A.

[20] Huber TA, Sornette D (2016). Can there be a physics of financial markets? Methodological reflections on econophysics. Eur. Phys. J. Special Topics.

[21] Mantegna RN, Stanley HE (2000). An Introduction to Econophysics.

[22] Rosenow B (2002). Fluctuations and market friction in financial trading. Int. J. Modern Phys. C.

[23] Yura Y, Takayasu H, Sornette D, Takayasu M (2014). Financial Brownian particle in the layered order-book fluid and fluctuation–dissipation relations. Phys. Rev. Lett..

[24] Yura Y, Takayasu H, Sornette D, Takayasu M (2015). Financial Knudsen number: Breakdown of continuous price dynamics and asymmetric buy-and-sell structures confirmed by high-precision order-book information. Phys. Rev. E.

[25] Kanazawa K, Sueshige T, Takayasu H, Takayasu M (2018). Derivation of the Boltzmann equation for financial Brownian motion: Direct observation of the collective motion of high-frequency traders. Phys. Rev. Lett..

[26] Kanazawa K, Sueshige T, Takayasu H, Takayasu M (2018). Kinetic theory for financial Brownian motion from microscopic dynamics. Phys. Rev. E.

[27] Rosser JB (2016). Entropy and econophysics. Eur. Phys. J. Spec. Top..

[28] Ponta L, Carbone A (2018). Information measure for financial time series: Quantifying short-term market heterogeneity. Physica A.

[29] Mandelbrot B (1963). The variation of certain speculative prices. J. Business.

[30] Mandelbrot B (1997). Fractals and Scaling in Finance: Discontinuity, Concentration, Risk.

[31] Mantegna RN, Stanley HE (1994). Stochastic process with ultraslow convergence to a Gaussian: The truncated Lévy flight. Phys. Rev. Lett..

[32] Koponen I (1995). Analytic approach to the problem of convergence of truncated Levy flights towards the Gaussian stochastic process. Phys. Rev. E.

[33] Tsallis C (1988). Possible generalization of Boltzmann–Gibbs statistics. J. Stat. Phys..

[34] Alonso-Marroquin F, Arias-Calluari K, Harré M, Najafi MN, Herrmann HJ (2019). Q-Gaussian diffusion in stock markets. Phys. Rev. E..

[35] Chaudhuri P, Berthier L, Kob W (2007). Universal nature of particle displacements close to glass and jamming transitions. Phys. Rev. Lett..

[36] Clara-Rahola J, Puertas AM, Sánchez-Granero MA, Trinidad-Segovia JE, de las Nieves FJ (2017). Diffusive and arrested like dynamics in currency exchange markets. Phys. Rev. Lett..

[37] Sánchez-Granero MA, Trinidad-Segovia JE, Clara-Rahola J, Puertas AM, de las Nieves FJ (2017). A model for foreign exchange markets based on glassy Brownian systems. PloS One.

[38] Scalas E (2006). The application of continuous-time random walks in finance and economics. Physica A.

[39] Meerschaert MM, Scalas E (2006). Coupled continuous time random walks in finance. Physica A.

[40] Ang A, Timmermann A (2012). Regime changes and financial markets. Ann. Rev. Finan. Econ..

[41] D’Amico S, King TB (2013). Flow and stock effects of large-scale treasury purchases: Evidence on the importance of local supply. J. Finan. Econ..

[42] Bauer MD, Neely CJ (2014). International channels of the Fed’s unconventional monetary policy. J. Int. Money Finan..

[43] Pearce DK, Roley VV (1983). The reaction of stock prices to unanticipated changes in money: A note. J. Finan..

[44] R.A. de Santis & W. Van der Veken. Forecasting macroeconomic risk in real time: Great and COVID-19 Recessions. *Eur. Central Bank Working Paper Series*. **2436**, 1 (2020).

[45] N. Fernandes. Economic effects of coronavirus outbreak (COVID-19) on the world economy. *IESE Business School Working Paper Series*. **WP-1240-E**, 1 (2020).

[46] Hamilton JD (1989). A new approach to the economic analysis of nonstationary time series and the business cycle. Econometrica.

[47] Bouchaud J-P, Gefen Y, Potters M, Wyart M (2004). Fluctuations and response in financial markets: The subtle nature of ‘random’ price changes. Quantit. Finan..

[48] Borysov SS, Balatsky AV (2014). Cross-correlation asymmetries and causal relationships between stock and market risk. PloS One.

[49] Tóth B, Lempérière Y, Deremble C, de Lataillade J, Kockelkoren J, Bouchaud J-P (2011). Anomalous price impact and the critical nature of liquidity in financial markets. Phys. Rev. X.

[50] Dhont JKG (1996). An Introduction to Dynamics of Colloids.

[51] Cutler DM, PoterbaPoterba JM, Summers LH (1988). What moves stock prices?. Natl. Bureau Econ. Res. Working Paper Series.

[52] Cornell B (2013). What moves stock prices: Another look. J. Portfolio Manag..

[53] MacKinlay A (1997). Event studies in economics and finance. J. Econom. Literature.

[54] Mahata A, Rai A, Nurujjaman M, Prakash O, Prasad Bal D (2020). Characteristics of 2020 stock market crash: The COVID-19 induced extreme event. Chaos..

[55] Rai, A., Mahata, A., Nurujjaman, Md. & Prakash, O. Statistical properties of the aftershocks of stock market crashes revisited: Analysis based on the 1987 crash, financial-crisis-2008 and COVID-19 pandemic. *Int. J. Mod. Phys. C*, 2250019. 10.1142/S012918312250019X.

[56] Evans DJ, Morriss GP (1990). Statistical Mechanics of Non-equilibrium Liquids.

