# Supplementary material for: Linear response theory in stock markets
Source: Sci Rep. 2021 Nov 29;11:23076. doi: 10.1038/s41598-021-02263-6 (PMC8630003; doi:10.1038/s41598-021-02263-6)
Supplement: Supplementary file 1 — Supplementary Information. [file 41598_2021_2263_MOESM1_ESM.pdf]

Linear response theory in stock markets: Supplementary Information

Antonio M. Puertas,[1] Juan E. Trinidad-Segovia,[2] Miguel A. Sánchez-Granero,[3] Joaquim Clara-Rahora,[4] and F. Javier de las Nieves[1]

[1] *Departamento de Física Aplicada, Universidad de Almería, 04.120 Almería, Spain*

[2] *Departamento de Economía y Empresa, Universidad de Almería, 04.120 Almería, Spain*

[3] *Departamento de Matemáticas, Universidad de Almería, 04.120 Almería, Spain*

[4] *KHN Capital Consulting, Avda.Diagonal 640 6th floor, 08017 Barcelona, Spain*

(Dated: November 1, 2021)

Abstract

In this supplementary information the analysis presented in the paper is applied to stocks from the New Your Stock Exchange (NYSE), and to a set of European stocks from different national floors. The results presented here provide further support to the conclusions drawn in the paper.

INTRODUCTION

The analysis presented in the manuscript has been performed on NASDAQ stocks. In order to provide further support to the conclusions, we extended the analysis to other stock markets. In all cases, we take the log-return as the variable conjugate to the external force, as concluded in the paper, and test the predictions for the evolution of the log-price and log-return after an event, and for the linear relationship between the log-return and log-price.

Two sets of stocks are considered. In the first case, the New York Stock Exchange (NYSE), the biggest market in terms both of capitalization and trade volume, is considered. In the second one, a set of European stocks from different national floors is conformed and studied. All data for both sets have been taken from Yahoo! Finance, with a time resolution of 1 day.

NEW YORK STOCK EXCHANGE

This set comprises 1084 stocks, from 2001 to 2020, and is comparable to the set of stocks from the NASDAQ.

In Fig. S1 the average response of the log-price and log-return after an event is presented, together with the calculations from the correlation functions, as studied previously for the set of stocks from the NASDAQ. Around 11000 events can be identified, ca. 5500 positive events and 5700 negative events. Following Figs. 1 and 2 in the paper, positive and negative events are averaged, and the absolute log-price and log-return variations are presented. However, different from the case of NASDAQ, the overshoot after the event in the log-price, and the subsequent relaxation is much less important (below 10%).

For the analysis of the log-price evolution, the integral of the log-return autocorrelation function (ACF) has been used. Again, the predicted response shows an almost unnoticiable relaxation to equilibrium, in agreement with the observed response. For the log-return, on the other hand, its normalized ACF $C(v^2, v^2)$ gives the response. The agreement of both quantities is comparable to the case of NASDAQ stocks, although less impressive due to the absence of memory.

Fig. S4 tests the relationship between the log-return change and log-price variation provoked by an event. The trend of the data is compatible with both of them being proportional

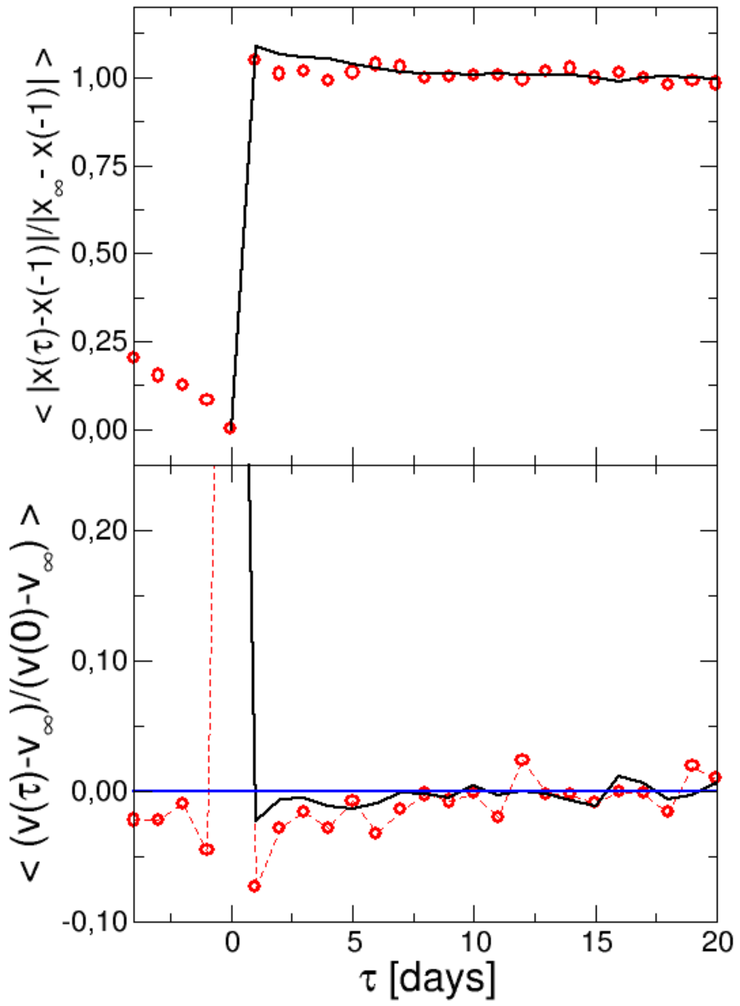

FIG. S1. Average response of the set of NYSE stocks after an event (symbols) and predictions from linear response theory (lines): Log-price is presented in the upper panel, and volatility in the lower one.

to the external force. The dashed line in the figure gives the prediction from the theory, $\Delta v_\infty = k_v/k_x \Delta x_\infty$, which describes approximately the experimental data close to the origin. The transport coefficients, as calculated within linear response are:

$$k_x = -\int_0^\infty \langle v(\tau)v(0)\rangle d\tau = -1.5 \cdot 10^{-3}$$

$$k_v = \langle v(0)v(0)\rangle - \lim_{\tau\to\infty} \langle v(\tau)v(0)\rangle = 2.6 \cdot 10^{-3}.$$

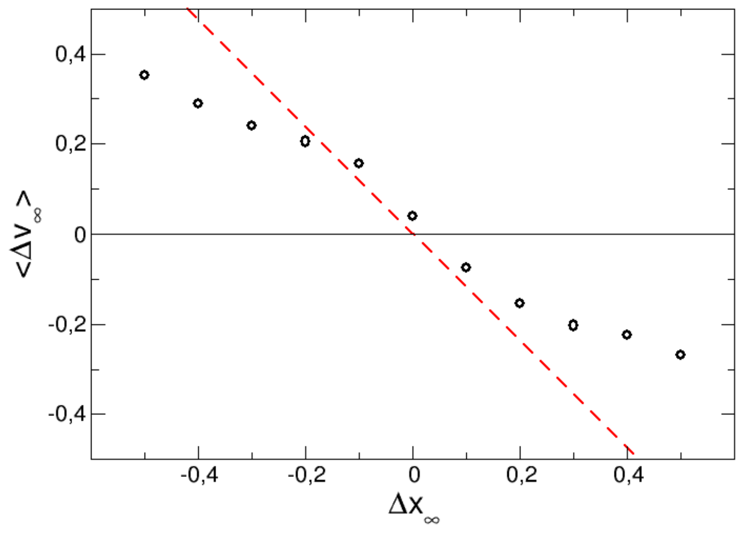

FIG. S2. Mean log-return variation as a function of the total log-price variation for the NYSE stocks. The daashed line is the prediction from the values of k_x and k_v.

EUROPEAN STOCKS

NASDAQ and NYSE are the world biggest markets in terms of capitalization and trade volume. Thus, in order to build a set of stocks of comparable characteristics we selected European stocks from companies that have belonged continuously to the national indices of the UK (FTSE100), Germany (DAX30), France (CAC40), Spain (IBEX35), Switzerland (SMI), Italy (FTSE MIB), Portugal (PSI20), and Holland (AEX). This set comprises 240 stocks, corresponding to big and stable European companies, sampled every day since 2010 to 2019.

In Fig. S3 the response of the log-price and log-return after an event are presented, together with the calculations from the correlation functions, as studied previously. To improve the statistics, events are defined with a different threshold: an event occurs for a given stock when the one day log-return is more than 2.5 times the root mean square deviation of log-returns of this stock. Even so, less than 600 events are identified, or less than three events per stock in ten years. Again, positive and negative events are averaged, and the absolute log-price and log-return variation are presented.

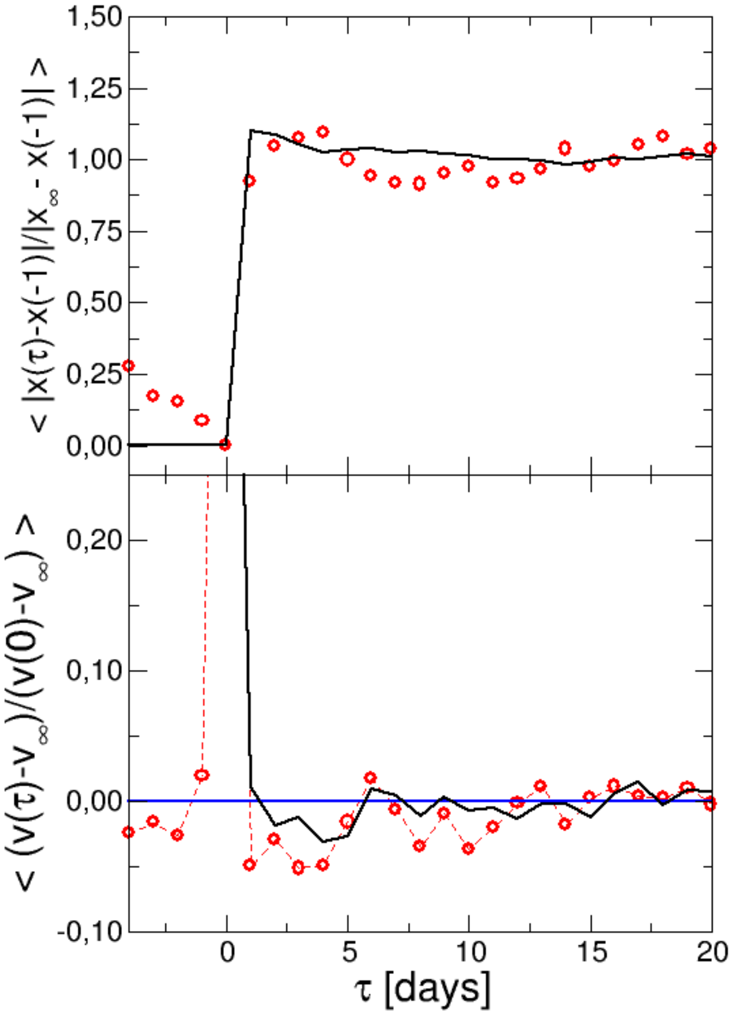

FIG. S3. Average response of the set of European stocks after an event (symbols) and predictions from linear response theory (lines): Log-price is presented in the upper panel, and volatility in the lower one.

In agreement with the theory expectations, the correlation function $C(v, v)$ gives the response of the log-return, and its integral corresponds to the response of the log-price. The agreement of both quantities is similar to the previous cases, although the noise of the data here is increased.

The relationship between the log-return change and log-price variation provoked by an event is studied in Fig. S4. The data, close to the origin, can be described by the straight line $\Delta v_\infty = k_v/k_x \Delta x_\infty$, with k_v and k_x calculated from LRT:

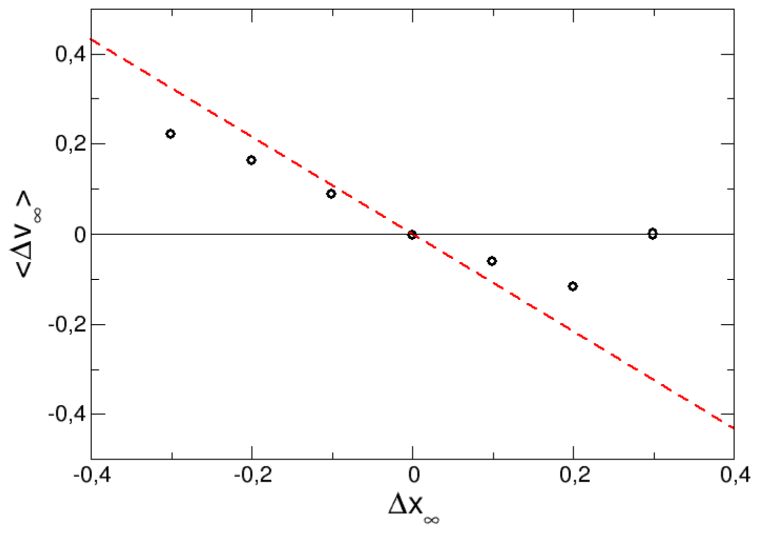

FIG. S4. Mean log-return variation as a function of the total log-price variation for the set of European stocks. The dashed line is the prediction from the values of k_x and k_v.

$$k_x = -3.1 \cdot 10^{-4} \qquad\qquad k_v = 3.6 \cdot 10^{-4}$$

Note that, due to poor statistics of this system, these data are strongly affected by numerical errors, despite the agreement shown in Fig. S4.
